# Data-Driven Modeling of the Bicalutamide Dissolution from Powder Systems

**DOI:** 10.1208/s12249-020-01660-w

**Published:** 2020-03-31

**Authors:** Aleksander Mendyk, Adam Pacławski, Joanna Szafraniec-Szczęsny, Agata Antosik, Witold Jamróz, Marian Paluch, Renata Jachowicz

**Affiliations:** 1 Department of Pharmaceutical Technology and Biopharmaceutics, Jagiellonian University Medical College, Medyczna 9 St, 30-688 Kraków, Poland; 2 Institute of Physics, University of Silesia, Uniwersytecka 4, 40-007 Katowice, Poland; 3 Silesian Center for Education and Interdisciplinary Research, 75 Pulku Piechoty 1a, 41-500 Chorzow, Poland

**Keywords:** artificial intelligence, dissolution modeling, multivariate modeling, multi-scale modeling, solubility enhancement

## Abstract

Low solubility of active pharmaceutical compounds (APIs) remains an important challenge in dosage form development process. In the manuscript, empirical models were developed and analyzed in order to predict dissolution of bicalutamide (BCL) from solid dispersion with various carriers. BCL was chosen as an example of a poor water-soluble API. Two separate datasets were created: one from literature data and another based on in-house experimental data. Computational experiments were conducted using artificial intelligence tools based on machine learning (AI/ML) with a plethora of techniques including artificial neural networks, decision trees, rule-based systems, and evolutionary computations. The latter resulting in classical mathematical equations provided models characterized by the lowest prediction error. In-house data turned out to be more homogeneous, as well as formulations were more extensively characterized than literature-based data. Thus, in-house data resulted in better models than literature-based data set. Among the other covariates, the best model uses for prediction of BCL dissolution profile the transmittance from IR spectrum at 1260 cm^−1^ wavenumber. *Ab initio* modeling–based *in silico* simulations were conducted to reveal potential BCL–excipients interaction. All crucial variables were selected automatically by AI/ML tools and resulted in reasonably simple and yet predictive models suitable for application in Quality by Design (QbD) approaches. Presented data-driven model development using AI/ML could be useful in various problems in the field of pharmaceutical technology, resulting in both predictive and investigational tools revealing new knowledge.

## INTRODUCTION

Active pharmaceutical ingredient (API) solubility and dissolution rate in water are crucial factors governing API bioavailability. Currently, about 40% of marketed API and around 90% of drugs in development can be classified as poorly soluble in water. It challenges formulation process and could lead to difficulties with successful therapy (1). Formulation development strategies of poorly soluble drugs include particle size reduction, crystal modification, addition of surfactants, preparation of solid dispersions, or lipid formulations (2). API dissolution profile in time is a result of complex interactions including a physical form of API, presence and chemical character of excipients in the formulation, and preparation process parameters as well.

Bicalutamide (BCL), a non-steroidal antiandrogen drug, exhibits aqueous solubility as low as 3.7 μg/mL and is well absorbed following oral administration (3,4), which classifies BCL to class II of the Biopharmaceutics Classification System (BCS) (5). Researchers reported in the literature improved dissolution of the powder systems with BCL by complexation with β-cyclodextrin (6,7), preparation of solid dispersions using solvent evaporation method (8–11), milling (12,13), hot melt extrusion (14,15), and formation of co-amorphous systems (16). Desired dissolution improvement was achieved by trial and error approach, applying various qualitative, quantitative composition, and preparation methods under different conditions. Pharmaceutical formulation development and optimization based on the better understanding of the process, API, and physicochemical properties of excipients was proposed by regulatory agencies in the most recent guidelines for industry (17,18). The reliable solution in such case is the development of the empirical models based on a broad characteristic of the process parameters and formulation composition. Construction of the decent quality predictive models could be therefore beneficial both from the practical and theoretical points of view.

To quantify and reveal such complex relationships, multivariate data analysis methods need to be employed. Among them, artificial intelligence machine learning (AI/ML) tools are suitable solutions due to their capability of automatic knowledge discovery and automated selection of critical variables (19). The AI/ML tools work directly on the available data building model without any assumptions with use of the supervised learning paradigm: known states of the system are related to their corresponding control parameters. The inference built by AI/ML system might be a “black box” hidden model—a typical example is artificial neural network (20), where there is no knowledge of how the answers of the system are inferred. There are also other types of models developed with AI/ML like fuzzy systems (21), which might be completely decomposed to the human-readable form. For the latter, a very promising solution is genetic programming belonging to the evolutionary computations and resulting in mathematical formulas as final models (22,23).

The objective of this work was to present two independent paths to develop predictive models: one based on the literature data and another on the in-house laboratory data only. Analysis of the developed models leads to a better understanding of the factors influencing the dissolution process of BCL from powder systems prepared using various methods. It was therefore our secondary objective to demonstrate how AI/ML tools are capable of finding hidden relationships within vast data sets and merging various types of information from different scientific domains into the one consistent model.

## METHODS

### Overall Workflow

**Fig. 1:**
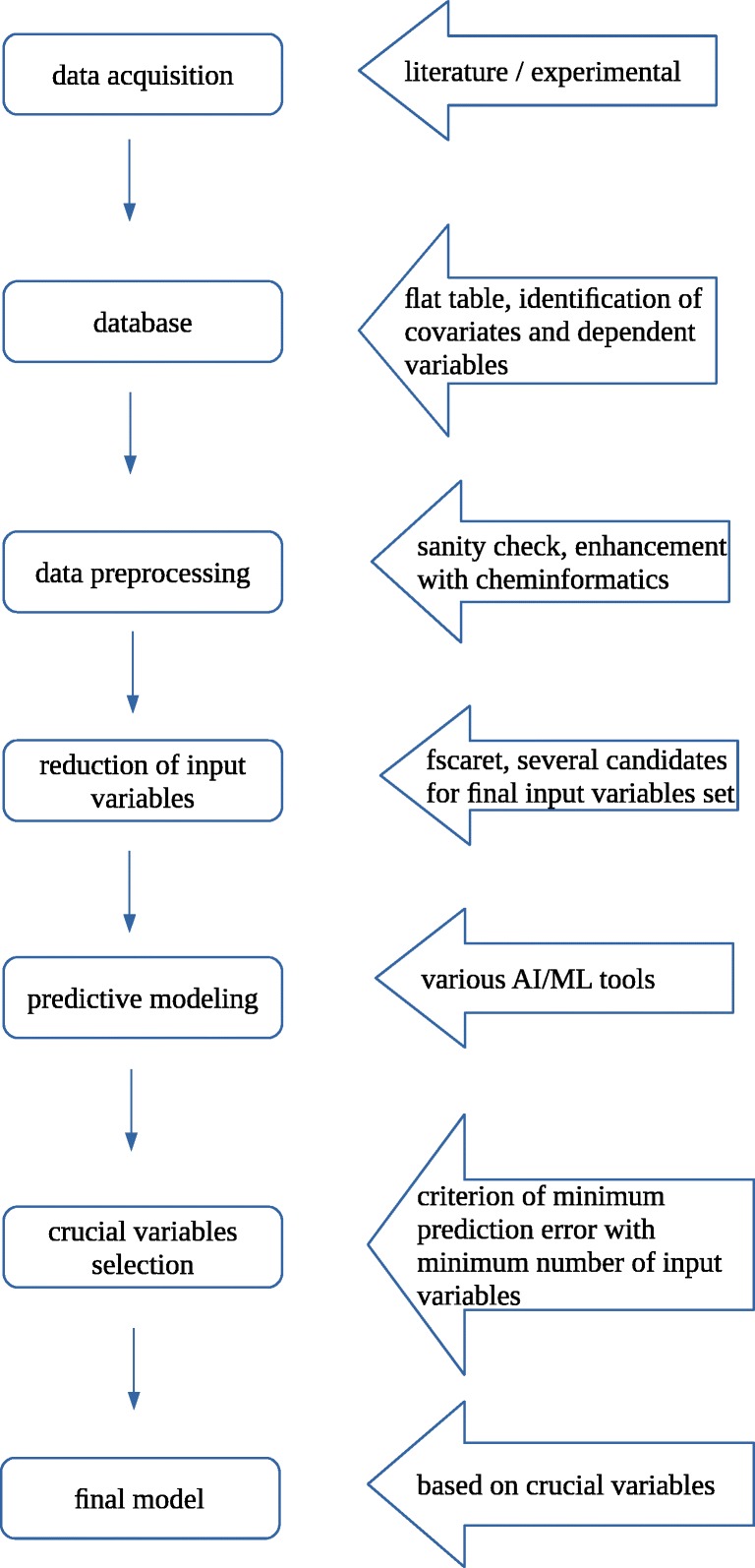
Workflow diagram

With empirical, data-driven approach for development of the model of bicalutamide dissolution from powder systems, we started our work with data acquisition stage followed by data preprocessing and modeling with feature selection stage (Fig. 1). Final models are built on the reduced input vector, namely crucial variable set.

### Data Sets

In the presented research, two independent data sets were prepared. The first one was built based on available literature data. The inclusion of a given formulation in the database was based on the availability of the following information found as essential for dissolution profile of powder systems:

- The qualitative and quantitative compositions of the powder system with bicalutamide as API,
- Preparation method of the formulation and its parameters,
- Conditions of the dissolution test,
- Availability of the complete dissolution profile in a tabular or graphical form.

The database consisted of 379 records for 51 powder systems with bicalutamide with each described by 204 input variables and one output variable representing the percentage of dissolved drug substance at the given sampling time. The structure of the data set is presented in Table I. The methods of the formulation preparation were encoded in the form of consecutive natural numbers and an additional variable, describing the process temperature expressed in kelvins. There were six different formulation preparation techniques encoded in the database: no method (pure API), evaporation method, physical mixture, kneading method, spray drying, and hot melt extrusion. The dissolution study was described by the type of applied apparatus, rotational speed of the paddle (USP Apparatus II), the percentage of sodium lauryl sulfate (SLS) added to the medium, volume, pH of the medium, and *in vitro* dissolution sampling time. Ten different excipients encoded with molecular descriptors were included into the database: colloidal silicon dioxide, β-cyclodextrin, 2-hydroxypropyl-β-cyclodextrin, hydroxypropylmethylcellulose (HPMC), lactose, microcrystalline cellulose (MCC), polyethylene oxide (PEO), sodium lauryl sulfate (SLS), and triethyl citrate.

**Table I:** Structure of the Literature-Based Data Set

Variable no.	Description
1	Content of excipient 1
2–98	Molecular descriptors of the excipient 1
99	Content of excipient 2
100–196	Molecular descriptors of the excipient 2
197–198	Formulation preparation method
199–204	Parameters of dissolution test
205	Dissolved amount of API after a particular time (%)

The second database was created based on in-house experimental data. Laboratory experiments involved the development of a set of binary and ternary powder systems with bicalutamide as an example of poorly soluble API. In comparison with the previous database, it was possible to extend formulation description by characteristics of the prepared formulations with differential scanning calorimetry (DSC) and infrared spectroscopy (IR). Chemical compounds were numerically encoded with molecular descriptors. Additionally, the description of polymeric excipients was enriched with their average molecular weight, and for inorganic compounds by the particle size (*D*_50_), specific surface area, and pore volume. A total of 36 variables described DSC results for each powder system. The IR spectra included in the database contained 3350 records representing the transmittance of the sample in the range of wavenumbers from 650 to 3999 cm^−1^. In total, the created database contained 3593 input variables and included 35 formulations for which the full characterizations were performed (DSC and IR results). The structure of the data set is presented in Table II.

**Table II:** Structure of the In-House Data Set

Variable no.	Description
1	Content of excipient 1
2	Average molecular weight of the polymer
3–100	Molecular descriptors of the excipient 1
101	Content of excipient 2
102–104	Additional characteristic of inorganic compounds: *D*_50_, specific surface area, and pore volume
105–202	Molecular descriptors of the excipient 2
203–238	DSC characteristic of the powder system
239–3588	IR characteristic of the powder system
3589–3592	Formulation preparation method characteristic
3593	Time
3594	Dissolved amount of API after a particular time (%)

### Molecular Descriptors

Excipients included in the individual powder systems were encoded with molecular descriptors defining their structural and physicochemical properties. The calculations were performed by MarvinSkech (ChemAxon, Hungary) (24). Descriptors for chemical compounds with low molecular weight were calculated based on an entire 3D model of the molecule. In the case of high molecular weight polymers and complex molecules (*e.g.*, HPMC, MCC), the calculation of the descriptors was carried out for a dimer of the representative fragment of a chain of polymer. In the case of polymers containing more than one type of building block, a dimer model was prepared for each of them. The final value of the descriptors took into account the molar ratio of the particular components and their substituents.

### Variable Selection

The size of provided input vectors may indicate its redundant nature, which may have a negative impact on the quality of the models and their generalization ability. The selection of variables, which are critical for a given problem, is an important step in the data-driven model development process. The analysis of variables’ importance was performed by using fscaret (25) package for the R environment (26), which allows estimating the significance of individual variables based on their performance in a set of models provided by the caret package. The software offers data preprocessing and an initial reduction of variable vector, based on a variance correlation matrix analysis. Besides, two measures of model performance are introduced: root mean squared error (RMSE) and mean squared error (MSE). In the current research, four separate computational experiments have been carried out to select significant variables, including the possibility of preprocessing data and both available model performance measurements.

### Modeling

Data sets with reduced input vector were split according to the 10-fold cross-validation method into the learning and testing files. Models were trained on the larger part of data (90%) and evaluated on the test data sets (10% of data). The latter resulted in the estimation of generalization error expressed as the average RMSE calculated over 10 repetitions of this procedure each time with different parts of data excluded to the test sets. In the presented work, we applied various computational tools for predictive modeling: rule-based systems, random forest, artificial neural networks, fuzzy logic, and genetic programming. Each one of the abovementioned tools processes data in the iterative manner, thus accounting for the training process where the actual model is built automatically on the provided data only.

### Rule-Based Systems

*Cubist* (27) package for R environment allows building models based on rule sets and linear regression equations. One hundred different models were constructed for every data set. Various models were created by modifying the parameter specifying the maximum number of rules in the model. Extrapolation and sample parameters had values of 100 and 0, respectively.

### Random Forest

The *randomForest* (28) package allows building decision tree models using the algorithm proposed by Leo Breiman (29). Approximately 2000 models have been built and tested for every input vector. The following set of parameters was modified in every model:

- Number of randomly sampled variables for each distribution was a set from 1 to 10,
- Number of end nodes in a given model took values from 10 to 1000,
- Count of trees in the model varied from 10 to 1000.

### Artificial Neural Networks

There were three different tools applied in the presented work employed for building artificial neural network (ANN) models. The first one *monmlp* (30) is based on expert committees composed of many individual artificial neural network models used for training nonlinear optimization algorithm—nlm(). The algorithm design provides fast adjustment of ANN weights. Approximately 1800 models were prepared for a single input vector to be modeled with the *monmlp* package. The individual model consisted of 2 hidden layers composed of a total of 4 to 50 neurons. Hyperbolic tangent and linear functions were applied as transition functions for artificial neurons in hidden layers and output layer, respectively. The number of iterations for individual computing tasks ranged from 10 to 1000. The second tool *neuralnet* (31) was designed to build multilayer artificial neural networks. The learning process was powered by classical backpropagation algorithm and various variants of the resilient backpropagation algorithms. A single model was constructed of 2 or 3 hidden layers, each containing from 3 to 30 neurons. Hyperbolic tangent (tanh) was used as an activation function. Two error measurements were applied: constant error (CE) and the sum of squared errors (SSE). Roughly 1000 models were built for each input vector. Another tool, the *h2o* package, was used for simulation of multilayered neural networks with many hidden layers, thus introducing deep learning approach. A single model was composed of 2 to 8 hidden layers and the total number of neurons varied from 4 to 438. Learning time was set from 1000 to 10^7^ iterations. The hyperbolic tangent was used as an activation function for all neurons in the ANNs. The total number of trained models exceeded 2500 for each input vector.

### Fuzzy Systems

The *fugeR* (32) package enables the development of fuzzy systems using genetic algorithms. The individual parameters describing the model structure and learning algorithms varied in the following way:

- Maximum number of rules (maxRules): 10–50,
- Maximum number of variables for each rule (maxVarPerRule): 1–20,
- Number of generations: 100–500,
- Population size: 50–500,
- Number of singletons for the output value (labelsMF): 5–15,
- Percentage of inherited chromosomes (elitism): 10–100%

The total number of created and tested models was over 2500 for a single input vector.

### Genetic Programming

The *rgp* (33) package enables the use of genetic programming (GP) methods to create models representing a given problem. During the simulated evolution process, *rgp* develops automatically various mathematical equations of which the most predictive ones constitute the final solution. The form of easy-to-read mathematical equation significantly simplifies their analysis. Two different evolutionary algorithms were applied:

- The generational evolutionary multi-objective optimization algorithm,
- Archive-based Pareto tournament multi-objective optimization algorithm.

Every computational experiment was divided into 100 steps of evolution, with 5,000,000 iterations each. Population size was set to 500. Maximum chromosome length defining the degree of complexity of the solution varied from 10 to 100. Additionally, three error measures were applied to determine the performance of models in the evolution process: root mean squared error (RMSE), mean squared error (MSE), and a sum of squared errors (SSE).

### *Ab Initio* Modeling

 ORCA software (34) was used for *in silico* modeling of IR spectra. Vibrational analysis was carried out using a density functional theory (DFT) on the B3LYP level with 6-311G basis set enhanced with one set of first polarization functions on all atoms (d,p) atom-pairwise dispersion correction, auxiliary valence triple-zeta basis set with “new” polarization functions (def2-TZVP), Becke-Johnson damping (D3BJ), and conductor-like polarizable continuum model (CPCM) with water as a solvent. Two-molecule systems were analyzed with the ORCA clustering approach. Before the DFT calculations, the geometries of all molecules were optimized in a two-stage approach: first by conformational analysis and then with the PM3 method. The XYZ files for ORCA were prepared with Marvin Sketch (24). BCL and two carriers, namely polyvinylpyrrolidone (PVP) and macrogol (PEG), were studied alone and as two-molecule systems (BCL + carrier). Due to the high demand of DFT for computational resources, PVP was presented as a dimer and PEG as oligomer equivalent to PVP dimer in the number of atoms. Results from ORCA simulations were analyzed with Avogadro open-source software, where spectrum area of interest was chosen and structural elements of chemical compounds responding to particular frequencies identified.

## RESULTS

### Models Built Based on the Literature Data Set

The analysis of the variables importance based on *fscaret* results allowed the reduction of the original input vector dimension. As a result, ten new input vectors containing from 7 to 24 input variables were given for further investigation. Afterwards, the database was split to generate pairs of learning-testing sets according to the procedure of 10-fold cross-validation, and the full dissolution profile of bicalutamide was treated as a single data block. The generalization error of the constructed models varied from 11.09 to 19.33. The best predictive model was created with *rgp* package using the genetic programming methods. RMSE error and *R*^2^ for the model were 11.09 and 0.85 respectively. The worst performing models obtained by multilayer artificial neural networks were created with *neuralnet*. Rule-based systems and random forest models performed well with RMSE error above 13.0 and *R*^2^ close to 0.80. The best model results in Eq. 1. It predicts the amount of bicalutamide dissolved after a given time (*Q*_t_) based on six input variables: quantitative composition of the powder system, three molecular descriptors of the excipient, preparation method, and time of measurement. The summary results of the best performing models for individual CI tool are presented in Table III. The final 6 input variables were the result of further input vector reduction performed by *rgp* tools on the 12-element input vector.

1 $$ {\displaystyle \begin{array}{c}Q\left[\%\right]=\mathit{\ln}\left[\left({X}_1\cdotp {X}_{12}+{X}_{12}+{C}_7\right)\cdotp \left({X}_3+{e}^{-{C}_8\cdotp {X}_2}\right)\right]\cdotp \\ {}\mathit{\ln}\left[{X}_1\cdotp {X}_{12}\cdotp \left(\left({X}_9-{C}_3\right)\cdotp \mathit{\ln}\left({C}_2\cdotp {X}_9\right)+{C}_1\right)\mathit{\ln}\left[{X}_9+{e}^{X_5-{C}_4}\right]+{C}_5\cdotp {X}_{12}+{C}_6\right]\end{array}} $$

where:

*C*_1_–*C*_7_: Constants
*X*_1_: Excipient 1 content (m/m%)
*X*_2_: Single bond count
*X*_3_: Smallest ring size
*X*_5_: Maximal projection radius
*X*_9_: Formulation method
*X*_12_: *In vitro* dissolution sampling time (min)
*Q*_t_: Bicalutamide dissolved after given time (%)

**Table III:** Performance of Predictive Models Builds Based on Literature Data and Selected 12-Element Input Vector

CI tool	RMSE	*R*^2^
Cubist	13.04	0.82
neuralnet	19.33	0.62
fugeR	15.43	0.75
h2o	14.32	0.77
rgp	11.09	0.85

### Models Built Based on In-house Data

The database created based on in-house data contained over 3500 input variables. On the basis of the ranking of variables created by the *fscaret *program, ten new input vectors including 7 to 24 crucial variables have been created. The prediction error of the models designed with different methods varied from 4.18 to 17.11 (Table IV). The worst performing models obtained with *neuralnet* package exhibited RMSE and *R*^2^ of 17.11 and 0.52, respectively. Models constructed with *fugeR* package show satisfactory predictions with RMSE = 10.89 and *R*^2^ = 0.81. The best model was obtained with genetic programming methods, and it is reflected in Eq. 2. Prediction error RMSE for the model was below 4.5, and *R*^2^ was over 0.95. The equation predicting the amount of dissolved bicalutamide uses six variables: quantitative composition of the formulation, IR transmittance of the sample at 1260 cm^−1^, preparation method, process temperature, number of cycles (in the case of the milling process), and *in vitro* dissolution sampling time. In the case of *rgp*, similarly for the literature data, the 20-input vector was automatically reduced to the abovementioned 6 crucial variables.

2 $$ {\displaystyle \begin{array}{c}Q\left[\%\right]={X}_1^{\frac{9}{16}}\cdotp \sqrt{X_{18}+{e}^{\mathit{\sin}\left(\mathit{\sin}\left(\sqrt{X_{17}}\right)\right)}}\cdotp {X}_{20}^{\frac{1}{8}}\cdotp {e}^{\frac{\mathit{\sin}\left(\mathit{\sin}\left({X}_{12}\cdotp \sqrt{X_{17}}\cdotp {X}_{20}-{C}_1\right)\right)}{16}}\\ {}{e}^{\frac{\mathit{\sin}\left(\mathit{\sin}\left(\mathit{\sin}\left({X}_{17}\right)\right)+{X}_1\cdotp {X}_{12}^2\right)}{16}+\frac{\mathit{\sin}\left({X}_{12}\cdotp {e}^{\mathit{\sin}\left(\sqrt{X_1}\cdotp {X}_{12}\right)}\cdotp {X}_{17}\right)}{16}+\frac{\mathit{\sin}\left(\sqrt{X_{17}}\right)}{4}+\frac{\mathit{\sin}\left({X}_1\cdotp {X}_{12}\cdotp {e}^{\mathit{\sin}\left({X}_{12}\right)}\right)}{16}}+\\ {}+{X}_{16}\cdotp {X}_{20}^{\frac{1}{4}}\cdotp {e}^{\frac{\mathit{\sin}\left(\sqrt{\sqrt{X_{18}}\cdotp \sqrt{X_{20}}+{e}^{\mathit{\sin}\left({X}_{12}\right)}+{X}_1}\right)}{2}+\mathit{\sin}\left(\sqrt{X_{17}}\right)}\end{array}} $$

where:

*C*_1_: Constant
*X*_1_: Excipient content
*X*_12_: IR transmittance of the sample at wavenumber 1260 cm^−1^,
*X*_16_: Preparation method
*X*_17_: Process temperature
*X*_18_: Number of milling cycles
*X*_20_: *In vitro* dissolution sampling time
*Q*_t_: Bicalutamide dissolved after given time (*X*_20_)

**Table IV:** Performance of Predictive Models Constructed Based on In-house Data Based on the 20-Element Input Vector

CI tool	RMSE	*R*^2^
Cubist	12.44	0.72
randomForest	11.21	0.79
h2o	13.13	0.70
neuralnet	17.11	0.52
rgp	4.18	0.97
fugeR	10.89	0.81

### Model Validation

**Fig. 2:**
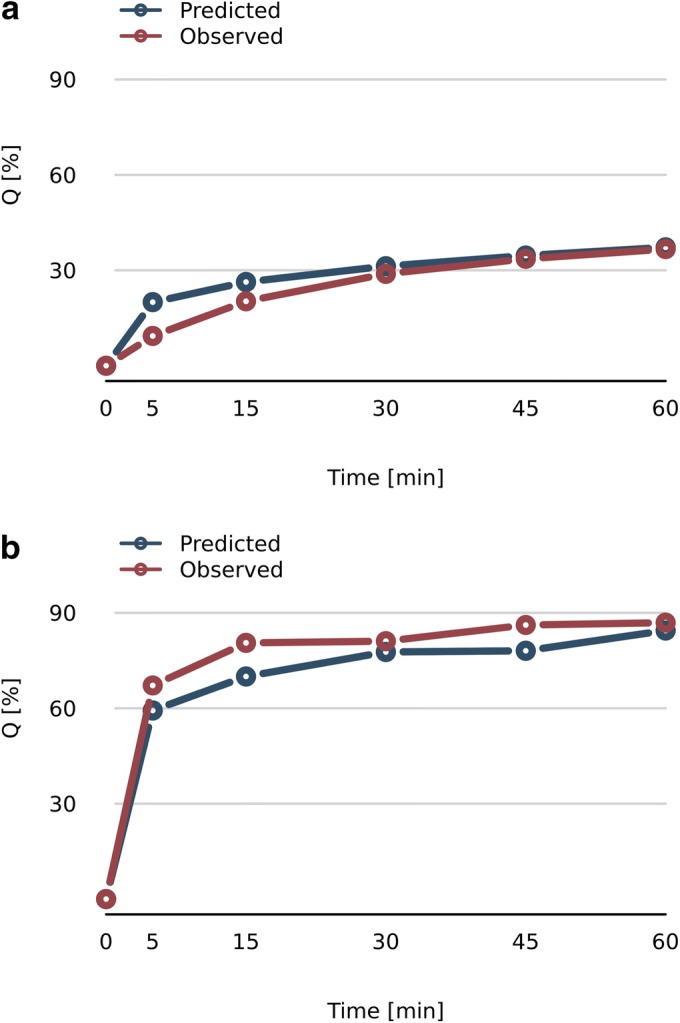
Predicted and observed dissolution profiles of BCL from various powder systems: **a** formulation composed of magnesium aluminometasilicate and BCL in 1:1 mass ratio and produced using evaporation process; **b** powder system composed of vinylpyrrolidone-vinyl acetate copolymer and BCL in 1:2 mass ratio and produced using milling process

After models were developed and tested according to the applied cross-validation method, additional powder systems were produced and characterized in the laboratory. Newly collected data were applied as a validation data set for the best model (Eq. 2), which delivered additional information about model predictive capability. The validation data set encoded 11 powder systems among which one record represented bicalutamide processed by the milling process, whereas ten records were binary powder systems with BCL and polymers or inorganic substances used as excipients. In total, 6 different excipients were used in the production process in which 3 (vinylpyrrolidone-vinyl acetate copolymer, colloidal silica, and magnesium aluminometasilicate) were not present in the original data set. Powder systems were produced using supercritical carbon dioxide, solvent evaporation, or milling process. RMSE and determination coefficient for the validation data set were equal to 9.89 and 0.92 respectively. Comparisons between predicted and experimental dissolution profiles were presented in Fig. 2a and b. Results presented in Fig. 2b refer to the powder system composed of vinylpyrrolidone-vinyl acetate copolymer and BCL in 1 to 2 mass ratio produced by the milling process. Dissolution profiles presented in Fig. 2a concern formulations prepared using the evaporation process and contained 50% of magnesium aluminometasilicate and 50% of BCL. It is noteworthy that both presented formulations contain excipients that were not present in the original database on which the model was developed and yet the model predicted properly whole dissolution profiles for BCL binary powder systems both with polymer and inorganic compounds used as excipients.

### *Ab Initio* Modeling

**Fig. 3:**
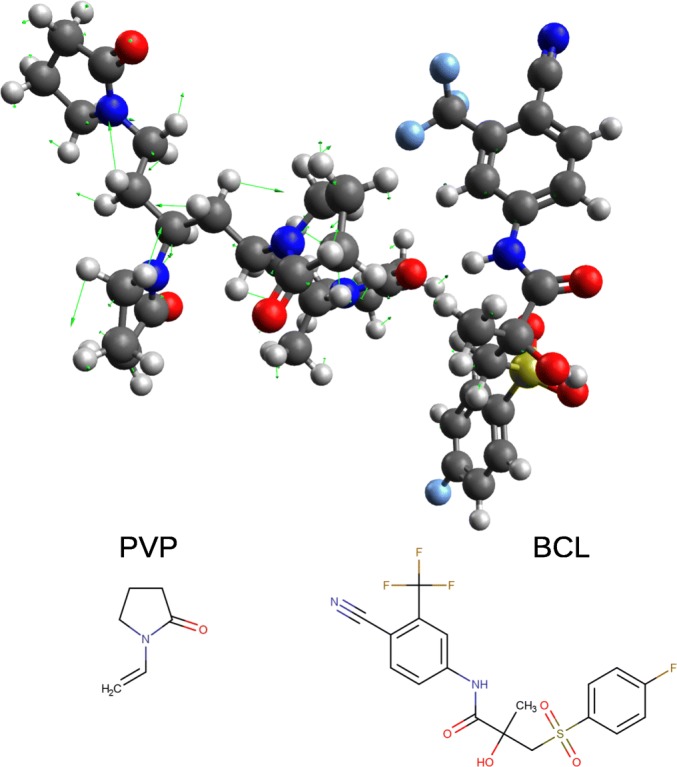
Results of vibrational analysis of BCL with PVP at the wavenumber of 1260 cm^−1^. Green arrows display force vectors

The studies with DFT modeling resulted in IR spectra calculated for several systems: BCL, PVP, PEG and BCL-PVP, BCL-PEG binary systems. Based on the wavenumber of 1260 cm^−1^ selected by our *rgp* model (Eq. 2), we analyzed the pattern of vibrations visualized with Avogadro software. An example of Avogadro-based visualization is presented in Fig. 3. When analyzing Fig. 3**,** it is evident that most of the vibrations at 1260 cm^−1^ are observed in the PVP chain.

## DISCUSSION

The comparative analysis of the models obtained for two prepared databases led to observation of the lower prediction errors for models created with in-house laboratory data. This is not surprising as multicenter data are usually characterized by a larger variability coming from different conditions of various laboratories and equipment. The best models for both databases were obtained using genetic programming methods. Mathematical equations developed automatically by *rgp* package predicted the amount of bicalutamide dissolved at a given time point with satisfactory accuracy. In both cases, models use six input variables and differ in the number of constants and structure. Both equations (Eq. 1 and Eq. 2) use quantitative composition of powder systems, information on the type of preparation method, and *in vitro* dissolution sampling time. According to the common knowledge in the field, this is a very sensible behavior of the AI/ML tools as they properly recognized variability in the qualitative and quantitative compositions, technological parameters of preparation of powder systems, and even the assay conditions. It is also noteworthy that since we used multiple input single output (MISO) modeling systems, then the time variable is essential to represent the whole dissolution profile as it is encoded in the database *via* several data records. The time variable was found as the most important input in both models. Despite the plethora of AI/ML tools used in this study, we were focused mostly on the equations developed by *rgp*. The main reason is that they turned out to be the most successful models in the task of prediction of BCL dissolution from binary systems. However, another point in favor of *rgp* is model transparency and relative simplicity in comparison with the random forests with thousands of decision trees or artificial neural networks with their hidden layers. The latter term was conceived to emphasize ANNs as black boxes. It is literally impossible to trace the decision path of fully trained ANN processing signals through their hidden layers. Transparency of a classical mathematical model provides means for its validation in the future—the procedure that is required from the pharmaceutical industry by every regulatory agency worldwide. In other words, all the above used AI/ML tools were employed from the empirical point of view to find out what might be the best possible predictability level in the analyzed problem and then, if possible, a tool with the most transparent, so-called white box, solution was chosen as the final one. This is the essence of a data-driven approach where the data and problem definition play a key role and the modeling tools are employed on the principle of the best possible predictability without prior assumptions of their mode and/or mechanisms of operation. Fortunately, it turned out that in our case the best models in terms of predictability were the most transparent ones.

Equation 1 developed for literature-based data employs three molecular descriptors calculated with the use of Marvin software to represent numerically excipients. Equation 2 does not include any of such molecular descriptors: it uses the information from IR spectrum at 1260 cm^−1^ instead. An important fact is that Eq. 2 was developed on our in-house data with the initial set of input variables containing both Marvin calculated molecular descriptors and measured IR spectra. However, no spectroscopic information was available for the data set based on literature. The feature selection procedure automatically discarded molecular descriptors in favor of spectroscopic information providing input vector with superior predictive ability when fitted to Eq. 2. Thus, the IR spectra used by our models could be regarded as a substitute to the molecular descriptors of excipients and employed empirically for a predictive model of BCL dissolution in water. It is another demonstration of a data-driven approach with autonomous decision ability of AI/ML tools. Moreover, it proves that AI/ML integrates smoothly various domains of knowledge into the consistent model even though technological properties and IR spectra seem to be far from each other as scientific fields. When faced with different types of data, AI/ML is capable of judgment of data relevance to the analyzed problem providing means for the detection of crucial variable sets tailored not only to the problem itself but to the available information about the analyzed problem as well. In order to justify and at least partially understand this decision of AI/ML, we performed *ab initio* modeling of chosen binary systems and their elements alone to simulate IR spectra and perform vibrational analysis with focus on the selected wavenumber 1260 cm^−1^. The latter is only loosely associated with C–F bonds and does not point to any other characteristic group (35). Since BCL contains four fluoride atoms, it is an indirect confirmation of possible interaction of BCL with carriers; however, no specific mechanism was revealed. Instead of looking for the particular molecular groups responsible for this wavelength selection, we observed the whole pattern of vibrations calculated by ORCA software. Looking through the results of vibrational analysis of BCL-PVP, BCL-PEG *vs.* BCL, and PVP and PEG alone, we found that no vibrations at 1260 ± 1 cm^−1^ were calculated either for BCL, PVP, or PEG alone. However, both binary systems, *i.e.*, BCL-PVP and BCL-PEG, exhibited such vibrational effects. This is therefore another indication of the interaction between BCL and carrier that AI systems found to be distinctive for prediction of dissolution rate of BCL. Moreover, when analyzing vibrational pattern of BCL-PVP *vs.* BCL-PEG, it was found that in the case of PCL-PVP, most vibrations occur in the carrier molecule, whereas for the BCL-PEG system, the BCL molecule was mostly responsible for the vibrational effect of the whole system. This corresponds to the behavior of solid dispersion systems described earlier by Szafraniec *et al*. (12), where complete amorphization of BCL was confirmed only for BCL-PVP solid dispersions due to the stabilizing effect of PVP over BCL. Bicalutamide unique behavior in the amorphous state was later explained by Rams-Baron *et al*. (36) with amide-imidic tautomerization effect. In none of the above in-depth physicochemical investigations of BCL amorphization, such IR wavenumber of 1260 cm^−1^ was identified as important for the observed phenomena. Thus, modeling with AI/ML tools turned out to be complementary to the classical, mechanistic analysis pointing out to the unforeseen spectroscopic region as relevant to the BCL dissolution pattern from solid dispersions. Additionally, it is noteworthy to mention that even though results of DSC analysis were initially introduced to the original data set, they were completely discarded in the final models. It leads to the conclusion that thermal analysis and its parameters, *e.g.*, glass transition point (*T*_g_), do not exhibit quantitative relationships with BCL dissolution patterns. This conclusion is consistent with the findings of Szafraniec *et al*. (10) of the mechanism responsible for promotion of dissolution rate and extent of BCL in BCL-poloxamer (PLX) solid dispersions, which is not based completely on amorphization but on the solubilizing effect of PLX over BCL as well. Since BCL-PLX systems were present in the analyzed data set, AI/ML decided to select crucial variables covering various physical mechanisms enhancing BCL dissolution from solid dispersions and it turned out to be IR wavelength at 1260 cm^−1^. From the practical point of view, the selected IR wavelength of 1260 cm^−1^ might be used as a diagnostic tool for solid dispersion performance. Therefore, spectroscopic assessment enables the means for in-line measurements employed for manufacturing process control. It goes alongside with the modern strategies of Quality by Design (QbD) and process analytical technologies (PAT), where the quality of the product is controlled during the manufacturing process; and based on predictive modeling, suitable corrective actions are taken to prevent manufacturing out-of-specification products. Use of IR spectra is feasible in such applications due to the speed of spectrum acquisition and capability of application in the solid state, thus suitable for many pharmaceutical unit processes. Although AI/ML models are developed in the iterative mode requiring substantial amount of time to process data, their further use does not suffer from such burden—the result is calculated instantaneously. Moreover, our mathematical formulas developed with GP could be processed by any regular IT/ICT infrastructure which makes their implementation simple and inexpensive. All the abovementioned features make the presented approach a suitable way of QbD implementation for future smart factories, where automated decision-making will be essential for effective manufacturing process.

## CONCLUSION

The presented work resulted in a comparison of predictive models for bicalutamide dissolution based on either literature or in-house experimental data. Not surprisingly, both sources of data resulted in different models with different crucial variables selected. The IR spectra available for in-house data were found useful to be surrogates of molecular descriptors in predicting BCL dissolution pattern. The use of IR spectra is promising for the in-line measurements suitable for the application of QbD/PAT strategies in manufacturing solid dispersions. Application of spectral information for predictive modeling of BCL dissolution pattern is possible due to the data-driven approach performed without mechanistic assumptions and therefore resulting in non-standard findings expanding current knowledge of key factors controlling observed physical phenomena.
